# Effects of Dietary Inclusion of Tannin-Based Supplements on Performance and Health of Yearling Beef Heifers Grazing Toxic, Endophyte-Infected Tall Fescue

**DOI:** 10.3390/vetsci12080749

**Published:** 2025-08-12

**Authors:** Miriam A. Snider, Cody E. Gruber, Robin A. Cheek, Tom Hess, Elizabeth B. Kegley, Jeremy G. Powell, G. Doug Hufstedler, J. Daniel Rivera, M. Shane Gadberry

**Affiliations:** 1 Southwest Research and Extension Center, University of Arkansas System Division of Agriculture, Hope, AR 71801, USA; msnider@uada.edu (M.A.S.); drivera@uada.edu (J.D.R.); 2 Livestock and Forestry Research Station, University of Arkansas System Division of Agriculture, Batesville, AR 72501, USA; thess@uada.edu; 3 Department of Animal Science, University of Arkansas System Division of Agriculture, Fayetteville, AR 72701, USA; cody.gruber@okstate.edu (C.E.G.); rc057@uark.edu (R.A.C.); ekegley@uark.edu (E.B.K.); jerpow@uark.edu (J.G.P.); 4 Department of Animal and Food Sciences, Oklahoma State University, Stillwater, OK 74075, USA; 5 Silvateam USA, New York, NY 10022, USA; dhufstedler@silavteam.com

**Keywords:** animal performance, ergot alkaloids, fescue toxicosis, plant secondary metabolites, serum metabolites, tannins, yearlings

## Abstract

This study evaluated dietary inclusion of tannin-based feed additives on heifer performance and physiological measures associated with fescue toxicosis and vaccine response. Forty-five yearling beef heifers (292.6 ± 29.0 kg) grazing toxic, endophyte-infected (E+) tall fescue pastures were assigned to 1 of 3 treatment groups: (1) soybean hulls (SH; *n* = 3 pastures), (2) SH and BX tannin-saponin additive (BX; 10.0 g heifer^−1^ d^−1^; *n* = 3 pastures), and (3) SH and ATX tannin-polyphenol additive (ATX; 5.0 g heifer^−1^ d^−1^; *n* = 3 pastures). Treatments were administered over an 84 d period. Data were analyzed using R and SAS 9.4 with treatment as a fixed effect, block as a random effect, and pasture as the experimental unit. Treatment effects were assessed at the pasture level. Average daily gain (ADG) of heifers receiving ATX (0.53 kg/d) and BX (0.49 kg/d) were increased relative to heifers receiving SH only (0.23 kg/d) between D56 and D84 (*p* = 0.07). There was no impact of treatment on other performance measures (*p* > 0.15) or on serum metabolite responses including bovine herpesvirus type 1 (BHV-1) titers (*p* > 0.15), prolactin (*p* = 0.97), haptoglobin (*p* = 0.26), and blood urea nitrogen (BUN; *p* = 0.38). Preliminary results show the potential for improved weight gain, suggesting further investigation with more experimental units is warranted.

## Simple Summary

Providing supplemental plant extracts as feed additives may improve the health status of cattle grazing toxic, endophyte-infected tall fescue. While supplemental plant extracts, such as tannins, have the potential to mitigate the impacts of ergot alkaloids, the extent of these benefits may vary depending on tannin type and concentration. The objective of this study was to evaluate two proprietary tannin-based feed additives, Silvafeed BX and ATX, against soybean hulls only in terms of animal performance and health responses in yearling beef heifers grazing toxic, endophyte-infected tall fescue pastures. The average daily gain and weight gain of heifers supplemented with the ATX and BX tannin additives were greater than that of heifers receiving soybean hulls only between day 56 and day 84 of the study. Other measures, such as hair coat scores, did not differ between treatments. There was no impact of treatment on heifer vaccine response or serum metabolite responses including prolactin, haptoglobin, and blood urea nitrogen. Preliminary results show the potential for improved weight gain when heifers are supplemented tannin-based feed additives. This suggests further research with more cattle and pasture replicates is needed.

## 1. Introduction

Tall fescue (*Schedonorus arundinaceus* (Schreb.)), more specifically Kentucky-31, is a cool-season perennial grass that is one of the most wide spread and heavily utilized forages in the United States [1]. Tall fescue commonly has a symbiotic relationship with a fungal endophyte (*Epichloë coenophiala*) [2,3] that confers productivity characteristics to the plant. These characteristics include improved insect resistance and drought tolerance, allowing for greater plant persistence and improved competitive ability [4,5,6]. Due to these positive attributes, as well as improved plant nutritive qualities, tall fescue has been heavily utilized in livestock grazing systems [1]. The positive characteristics conferred to tall fescue are due to the production of ergot alkaloids (EA) by the fungal endophyte. While EA production is beneficial to the plant, consumption by livestock can be detrimental to animal health by leading to bovine fat necrosis, fescue foot, and fescue toxicosis. Although fat necrosis and fescue foot are detrimental to health and productivity, fescue toxicosis is of greatest concern to beef producers due to large economic losses that are incurred [7,8].

Due to the consumption of EA, cattle grazing toxic, endophyte-infected (E+) tall fescue during the spring grazing season may experience compromised growth performance, poor health, compromised immune function [9], and reduced dry matter intake (DMI). Ergot alkaloids function by binding to serotonergic and adrenergic receptors within the vascular system [3]. By binding to vascular receptors, EA leads to vasoconstriction of blood vessels, reducing blood flow to both organs and peripheral tissues [10]. Additionally, vasoconstriction of blood vessels disrupts thermoregulation by elevating body temperature, making cattle vulnerable to heat stress [3,10]. This is further exacerbated by persistently low serum prolactin concentrations which results in continued hair coat growth, leading to a delay in hair coat shedding well into the summer grazing season [11].

Numerous management strategies, products, and compounds have been evaluated to reduce the negative impacts of tall fescue toxicosis but many, such as pasture renovation, have been impractical or unsuccessful in mitigation of the aforementioned impacts [7,11]. Several studies have evaluated inter-seeding of legumes with E+ tall fescue to dilute the effects of EA with variable responses. Inclusion of red clover (*Trifolium pratense*) and white clover (*Trifolium repens*) has been shown to be an effective strategy to increase animal performance when cattle are grazing E+ tall fescue [11,12,13]. Improved performance when grazing inter-seeded pastures may be due to the presence of plant secondary metabolites (PSM) such as isoflavones and tannins. However, improvement in performance may not be a reflection of PSM but rather a higher plane of nutrition, particularly in relation to increased protein and energy, and improved intake [13]. Additionally, while the inclusion of clovers with E+ tall fescue may be effective, it may not always be an economically feasible strategy. Therefore, there remains a need to identify a practical strategy for mitigating tall fescue toxicosis that is both practical and economically feasible.

Supplying supplemental plant extracts, such as tannins, as feed additives may improve the health status of cattle grazing E+ tall fescue. Modes of action of supplemental plant extract additives may vary but often include antioxidant properties, improved utilization of nutrient supply, and non-selective binding that reduces alkaloid absorption and metabolism [11,14]. As previously mentioned, tannins are polyphenolic compounds that are synthesized by legumes [14,15] and are frequently utilized in ruminant diets [16]. They can be classified as hydrolysable tannins (HT), such as those found in chestnut leaves (*Castanea* spp.) or condensed tannins, such as those found in quebracho (*Schinopsis* spp.; CT) [16]. Tannins may alleviate the effects of tall fescue toxicosis as they have a high binding affinity for nitrogenous compounds, such as those found in EA [11]. This binding capability could reduce the toxic effects of EA by reducing the absorption of alkaloids through the gastrointestinal epithelia [17,18]. Villalba et al. [17] indicated that performance and intake of lambs receiving E+ tall fescue seed improved when supplemented with the tannin-containing legume sainfoin (*Onobrychis viciifolia*) when compared with a non-tannin-containing legume. However, sainfoin was not offered as a plant extract product nor was tall fescue grazed in this study.

Studies have shown that tannins may improve final body weight (BW), dry matter intake (DMI), and average daily gain (ADG) [16,19]. Additionally, tannins have been shown to possess properties demonstrating antimicrobial and antiparasitic effects [20,21] although impacts on health are highly variable based on the origin and type of tannin utilized [21]. While tannins have the potential to mitigate the post-ingestive impacts of EA, the extent of these benefits may vary depending on alkaloid structure, rumen and abomasum pH, and tannin concentration [11,22]. For example, structural differences between HT and CT may influence their ability to bind and neutralize EA, with research suggesting that CT form more stable complexes with alkaloids due to their higher molecular weight and greater affinity for protein-like compounds. Excessive inclusion of dietary tannins may be detrimental by decreasing palatability and DMI, which can decrease animal productivity and feed efficiency [22]. This presents challenges in determining the correct concentration and type of tannin to include to mitigate the effects of tall fescue toxicosis while simultaneously avoiding potential negative impacts from excessive dietary tannin inclusion. The objective of this study was to assess the dietary inclusion of two proprietary tannin-based feed additives compared to soybean hulls only on animal performance and physiological measures of yearling beef heifers grazing E+ tall fescue pastures.

## 2. Materials and Methods

### 2.1. Animal Care and Use

This project was approved by the University of Arkansas Division of Agriculture’s institutional animal care and use committee (#24025) and was conducted at the Livestock and Forestry Research Station (LFST; 35.826683N, 91.774796W) near Batesville, Arkansas from early April to late June 2024.

### 2.2. Weather Conditions

As previously described by Gadberry et al. [23], the LFST is an official reporting station for the National Weather Service (Station ID USC00030458). Additionally, the LFST has a weather station that is part of the Climate Reference Network. National Weather Service temperature (°C) and rainfall (mm) data for the year (yr) 2024 and historical data (1942–2023) were available through the National Oceanic and Atmospheric Administration climate data online portal (https://www.ncei.noaa.gov/access/past-weather/72501 (accessed on 30 May 2025)).

### 2.3. Animal Management

A total of 45 yearling beef heifers of primarily Angus background grazed E+ tall fescue as a single group prior to the start of the treatment period in spring 2024. Before assignment to treatment pastures, heifers were evaluated and confirmed negative for persistent infection with bovine viral diarrhea virus (PI-BVD) based on an ELISA antigen capture test (Cattle Stats, Oklahoma City, OK, USA). Cattle were placed on assigned study pastures 3 d prior to the beginning of the study and were stratified by hair coat color and body weight (BW). Pastures were composed of a gravelly silt loam soil type as described by Diaz et al. [24]. All replicate pastures were provided a trace mineralized salt block (United Salt Corporation, Houston, TX, USA) as well as artificial or natural shade and water pumped from a well into water tanks (MiraFount, Miraco Livestock Water Systems, Grinnell, IA, USA). Upon assignment to pastures, fecal samples were collected and submitted to the University of Arkansas System Division of Agriculture Parasitology lab for strongyle egg counts (FEC). After samples were collected for FEC, heifers were given an oral anthelmintic (Safeguard, Merck Animal Health Intervet, Inc., Rahway, NJ, USA) at the beginning of the study (D0) as well as a trace mineral injection dose (Multimin 90, Multimin USA, Inc., Fort Collins, CO, USA). Fecal egg counts were assessed again at the conclusion of the study (D84). Fecal samples utilized for FEC were processed as described by Gadberry et al. [23].

### 2.4. Experimental Treatments, Pastures, and Forage Management

Nine experimental pastures were located within 3 blocks with 3 pasture replicates per treatment. As described by Diaz et al. [24], pastures were previously fertilized with 60 kg/ha N prior to grazing. Each 1.62-ha pasture was stocked with 5 heifers. Pastures were sampled at the beginning of the study (D0) and every 28 days (d) until the conclusion of the study for forage mass and nutritive value (University of Arkansas Agricultural Diagnostics Laboratory, Fayetteville, AR, USA). At a study mid-point (D42), pastures were assessed for species composition using a step-point method to ensure pastures were primarily composed of E+ tall fescue. Briefly, the percentage of fescue in pastures was approximated by traversing pastures in a zig-zag pattern. Plant species were recorded at the point of the shoe at every fifth step. Additionally, pastures were evaluated for endophyte infection by collecting 20 fescue tillers/pasture and analyzing via an *Epichloë*-specific immunoblot assay (Agronostics Ltd., Watkinsville, GA, USA) with Kentucky bluegrass (*Poa pratensis*), annual ryegrass (*Lolium multiflorum*), cheatgrass (*Bromus tectorum*), and orchardgrass (*Dactylis glomerata*) as negative controls.

Treatments were offered over an 84 d period between early April and late June 2024. All heifers grazed primarily E+ tall fescue pastures for the duration of the study. Experimental treatments included: (1) soybean hulls (SH; control; *n* = 3 pastures), (2) SH and BX tannin-based feed additive (10.0 g heifer^−1^ d^−1^; BX; *n* = 3 pastures), and (3) SH and ATX tannin-based feed additive (5.0 g heifer^−1^ d^−1^; ATX; *n* = 3 pastures). Additive inclusion levels were determined based on product and company recommendations. Treatments were offered on an as-fed basis (1.8 kg/heifer) between 0800 and 0900 daily and fed via trough within each pasture. Each week (wk), BX and ATX additives were prepared in 3 batches with the BX and ATX additives blended with SH prior to feeding. The BX additive (Silvateam S.p.A, San Michele Mondovì, Italy) was composed of a blend of quebracho (*Schinopsis lorentzii*) and chestnut (*Castanea sativa*) tannins as well as mixed screenings selected for their specific saponin contents. The ATX additive (Silvateam S.p.A, San Michele Mondovì, Italy) was composed of a blend of quebracho and chestnut tannins as well as mixed screenings selected for their polyphenolic-rich profiles.

### 2.5. Animal Performance and Health Response Measures

Heifers were individually weighed every 28 d prior to morning feeding. Additionally, hair coat scores, fecal staining scores, fecal samples (25 g) for moisture content, and rectal temperatures were collected every 28 d. Fecal samples for moisture content were dried at 50 °C for 96 h in a forced air oven. Rectal temperature was recorded (GLA M900, Tech Instrumentation Inc., Elizabeth, CO) following temperature reading stabilization for at least 10 s. Hair coat scores (Table 1) and fecal staining scores (Table 2) were recorded by 2 trained observers with the average score between observers used for treatment comparisons. Average daily gain and weight gained/period were calculated at the end of the study.

Jugular blood samples were collected on D56, D70, and D84 of the study with an 18G needle into a 10 mL red-top vacutainer (BD, Franklin Lakes, NJ, USA). Samples were placed on ice until processing. Following blood collection on D56, heifers received a subcutaneous injection of a modified live multivalent bovine respiratory disease (BRD) vaccine (One Shot, Zoetis, Kalamazoo, MI, USA) including strains to protect cattle against respiratory disease caused by infectious bovine rhinotracheitis (IBR) virus, parainfluenza3 (PI3) virus, and bovine respiratory syncytial virus (BRSV); respiratory disease and viremia caused by bovine viral diarrhea (BVD) Type 1, including 1b, and Type 2 virus; and bovine pneumonia caused by *Mannheimia haemolytica* type A1. At processing, blood samples were allowed to clot at room temperature and centrifuged for 20 min at 1700× *g*. Serum was collected and stored at −20 °C until analysis. Samples corresponding to D0, D14, and D28 post-vaccination were processed and submitted to the Iowa State Veterinary Diagnostics Lab for evaluation for infectious bovine rhinotracheitis (bovine herpesvirus type 1; BHV-1) virus neutralizing (VN) antibody titers. Serum titers were reported as the log_2_ of the greatest serum dilution of serum that provided complete protection of cells with the lowest dilution tested being 1:4 and the greatest 1:256. Serum samples with a neutralization value of <2 were considered negative and samples ≥2 were considered positive. Additional serum collected on D56 was analyzed for blood urea nitrogen (BUN) using a colorimetric kit (TECO Diagnostics, Anaheim, CA, USA). Assays were performed in a flat-bottom 96-well plate. Samples were analyzed in duplicate on a microplate reader (Biotek EPOCH, Biotek Instruments Inc., Winooksi, VT, USA) at a λ of 340 nm. Additional serum samples collected on D14 post-vaccination were analyzed for haptoglobin concentrations using a commercial ELISA kit (Immunology Consultants Lab, Tigard, OR, USA) with samples analyzed in duplicate. Blood drawn on the final d of the study (D84) was analyzed for serum prolactin using a radioimmunoassay (RIA; University of Tennessee, Knoxville, TN, USA). The intra- and inter-assay coefficients of variation were 4.51% and 9.18%, respectively.

During the D28 collection timepoint, a BX treatment heifer was observed to be unthrifty with loose stool and poor weight gain. This heifer was noted as having a high FEC and treated with a coccidiostat (Corid 9.6% Oral Solution, Huvepharma, Peachtree City, GA, USA) and anthelmintic (Dectomax, Zoetis Inc., NJ, USA) and removed from the study based on study protocols. To assess the impact of removal, analyses were conducted with and without the affected animal. Results remained consistent, indicating minimal effect on overall treatment outcomes. An ATX treatment heifer was never observed visiting the bunk following supplemental feed delivery. This was not accompanied by any signs of illness or stress and was considered to be an outlier. With 14 out of 15 ATX treatment heifers readily appearing at the trough each feeding and heifers consuming the full portion of provided supplement, we do not believe there is any evidence to suggest that this response is attributed to the ATX feed additive. Further, statistical analysis with and without this heifer showed no impact on results.

### 2.6. Statistical Analysis

Linear mixed models were constructed and analyzed using the LME4 package for R. Measured responses were plotted to determine if any outlier data points were evident. Following raw data assessment, response values were aggregated to the pasture level prior to analysis as pasture was the experimental unit. When appropriate, the initial observation on D0 was included as a covariate and pasture location block was included as a random effect. The Kenward–Rogers method was used for estimating denominator degrees of freedom for ANOVA using the lmerTest package for R and pairwise comparisons using the emmeans package for R. Assumptions for normality, homogeneity, and independence were assessed where appropriate for all models with transformations applied when assumptions were not met. A log transformation was applied to BHV-1 titer data prior to analysis and titers were aggregated to the pasture level using the geometric mean instead of the arithmetic mean prior to further statistical analyses. The FEC was tested for normality using the Shapiro–Wilk test. Kendall’s tau was calculated between observers across all sample dates for both hair coat scores and fecal staining scores. Confidence intervals for historical temperature and precipitation data between 1942 and 2023 were evaluated using the MEANS procedure of SAS 9.4 (SAS Inc., Cary, NC, USA). Weight gain, serum prolactin, BUN, and haptoglobin measures were evaluated using the MIXED procedure of SAS 9.4 with treatment as a fixed effect and block as a random effect. Haptoglobin data was log-transformed after analysis for normalcy. Data were considered significant at *p* ≤ 0.10 with tendencies recognized at 0.10 < *p ≤* 0.15. Due to limited sample size, these thresholds were used to identify potential trends that might warrant further investigation.

## 3. Results and Discussion

This work evaluated the effects of supplementation of two proprietary tannin-based feed additives (BX and ATX) compared to SH only on the performance and health responses of yearling beef heifers grazing E+ tall fescue pastures. Research evaluating the impacts of tannins as a potential mitigation strategy for tall fescue toxicosis are lacking, indicating a need for further research. As a limitation of experimental design, a negative control (no SH) was not utilized in this study.

### 3.1. Weather Conditions and Climate 

**Figure 1:**
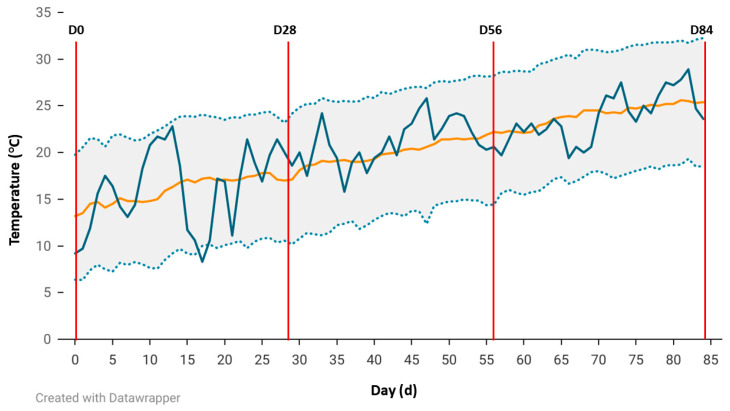
Daily average temperatures at the Livestock and Forestry Research Station beginning April 5 (D0) and ending June 28 (D84; blue) including historical averages (1942–2023; orange) and historical 95% confidence intervals (CI; 1942–2023, gray). Weigh days (D0, D28, D56, and D84) are designated in red.

**Figure 2:**
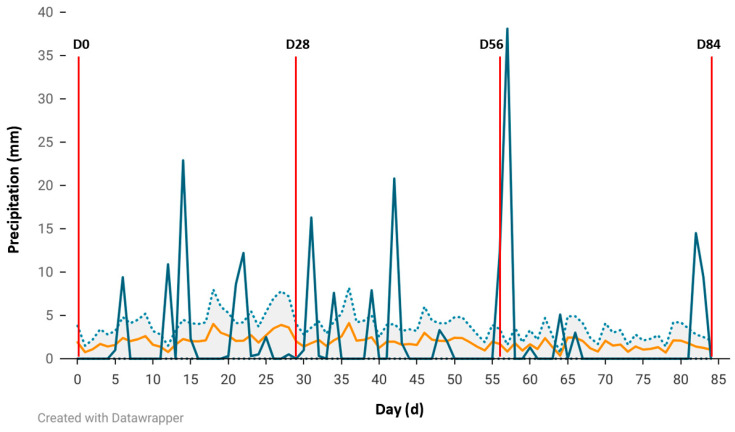
Total daily precipitation at the Livestock and Forestry Research Station beginning April 5 (D0) and ending June 28 (D84; blue) including historical daily averages (1942–2023; orange) and historical 95% confidence intervals (CI; 1942–2023, gray). Weigh days (D0, D28, D56, and D84) are designated in red.

Weather conditions are illustrated in Figure 1. (average daily temperature) and Figure 2. (daily precipitation). Both figures show historical data and data collected between 5 April and 28 June 2024, the respective starting and ending dates of the 84 d treatment period.

The average temperature for the 84 d treatment was 20.3 °C. Additionally, the cumulative total precipitation across the 84 d treatment period was 216.2 mm with an average monthly cumulative precipitation of 72.1 mm. Temperatures trended towards the lower temperature range during the first few wk of the treatment and grazing period before steadily increasing during the treatment period. Precipitation was generally negligible towards the end of the treatment period after D65. However, the precipitation events that occurred during the treatment period were generally above historical average precipitation with the greatest amount of precipitation occurring on D57 (38.1 mm).

### 3.2. Forage Mass, Forage Nutritive Value and Fescue Endophyte Infection

#### 3.2.1. Forage Mass and Forage Nutritive Value

Forage mass and E+ tall fescue nutritive quality are reported in Table 3.

Forage mass did not differ based on treatments for D0 (*p* = 0.78) and D84 (*p* = 0.72). However, forage mass was numerically less at the beginning of the experimental period and increased as the season progressed. Average forage mass throughout the treatment period tended to be greater for BX treatment pastures (2278.0 kg/ha) as compared to SH treatment pastures (2153.0 kg/ha) and ATX treatment pastures (2165.0 kg/ha; *p* = 0.12). A reduction in forage intake in cattle grazing toxic E+ tall fescue was to be expected. Although EA and field toxicity changes were not assessed throughout this study, increased temperatures and diminished forage quality could lead to decreased heifer performance as evidenced by the period-by-period reduction in heifer weight gain in the SH treatment group which will be discussed in further detail in subsequent sections. Additionally, it is possible that a decrease in intake may be due to dietary tannin inclusion. Tannins have been shown to reduce methane, allowing for a shift towards increased energy efficiency, similar to the action of ionophores such as monensin [24].

In general, forage nutritive quality did not differ significantly between treatment groups with a few exceptions. Mean (*p* = 0.14) and D84 (*p* = 0.14) crude protein (CP) content tended to be greater in BX treatment pastures as compared to ATX treatment pastures. The average CP and D84 CP did not differ between the SH experimental pastures and the pastures assigned to the BX and ATX treatments. It is possible that inclusion of the BX additive on pastures with a greater CP may have supported the trend for greater ADG and weight gain seen in heifers assigned to the BX treatment pastures. This may also contribute to the tendency for mean forage mass of BX pastures to be greater than that of SH and ATX supplemented pastures as greater CP levels can contribute to improved feed efficiency with cattle gaining more weight with less feed [26]. Neutral detergent fiber (NDF) content was less for BX treatment pastures on D0 (*p* = 0.06) but was equivalent to both ATX and SH treatment pastures at the end of the treatment period (*p* = 0.12). However, NDF tended to be greater for ATX treatment pastures (61.7% DM) compared to SH treatment pastures (59.3% DM) on D84 (*p* = 0.12). The average NDF content tended to be greater for ATX treatment pastures compared to that of SH and BX treatment pastures (*p* = 0.14).

#### 3.2.2. Tall Fescue Endophyte Infection

When experimental pastures were evaluated for species composition, they were found to be primarily composed of tall fescue (76.0 ± 11.0%). Pasture species composition did not differ among treatments (*p* = 0.95). Fescue endophyte infection rate averaged 96.0 ± 4.6% and did not differ among treatments (*p* = 0.98). This was similar to the endophyte-infection shown in a study by Poudel et al. [27] who indicated that their experimental E+ tall fescue pasture was 100.0% endophyte-infected with a total concentration of EA exceeding 1500 ppb across the 3-yr study. Based on our results, pastures were sufficiently toxic to induce fescue toxicosis as research has shown that animals grazing E+ tall fescue with an infection level exceeding 22.0% will display visible signs of fescue toxicosis [28]

### 3.3. Fecal Egg Counts

Studies have shown that tannin-containing forages or feeding tannin-based feed additives may control gastrointestinal parasites. Most research relating to tannins as a method for parasite control has focused on small ruminants [29,30]. However, other studies focusing on cattle have also shown a reduced parasite burden when tannins are included in the diet [31,32]. Fecal egg counts at the conclusion of the supplementation period were low with 48.0% of heifers having no strongyle eggs present in feces sampled. Among the heifers with positive FEC, the internal parasite load was minimal with egg counts averaging 10.7 EPG. Results showed that FEC was not normally distributed and was highly variable (66.0 ± 9.7 eggs/g (EPG)). Low and variable EPG suggests that parasite load among treatments was not sufficient to determine if the tannin-based feed additives (BX and ATX) would suppress internal parasite burden below the control treatment (SH).

### 3.4. Heifer Performance

Research has shown that cattle efficiency parameters and ADG associated with tannin-supplementation in both high-grain [33] and forage-based diets [19,34] may be improved. This is thought to be due to the changes in fate of dietary N [35,36,37]. However other studies have reported no impacts of dietary tannin inclusion on growth performance parameters [38]. Additionally, research related to cattle performance in response to tannin inclusion when grazing E+ tall fescue is limited with most studies conducted in sheep [14,17] or with tannins fed as a forage rather than as a feed additive [13,28]. In the current study, heifers assigned to SH treatment pastures tended to weigh less (287.1 kg) than heifers assigned to BX (294.4 kg) and ATX (296.6 kg) treatment pastures on D0 (*p =* 0.12; Table 4).

However, this weight difference cannot be attributed to tannin or SH supplementation as animals were weighed before supplementation began. Weight did not differ for the remainder of the treatment period. Average daily gain did not differ among treatments over the 84 d study. Numerical differences in ADG increased over time with the last 28 d period showing that ATX and BX supported greater weight gain than SH alone (*p* = 0.07). Furthermore, ADG and weight gained per period was numerically greatest for heifers receiving the ATX treatment as compared to the BX treatment. This indicates that the concentration of tannin-based feed additive included in the diet was not detrimental to animal intake as high levels of tannins often results in a DMI decrease [21,39]. However, this cannot be definitively concluded as DMI was not evaluated in this study. In a 3 yr study conducted by Poudel et al. [27], steers grazing primarily E+ tall fescue that were supplemented with tannin-containing *Sericea lespedeza*-based pellets tended to have greater ADG (0.60 kg/d) compared to steers supplemented with *Sericea lespedeza*-based pellets with polyethylene glycol (0.48 kg/d; *p* = 0.09). However, it should be noted that ADG was reported as a single average across the 3-yr study rather than calculated between periods. In the current study, weight gain (kg/period) data generally supports the identified trends within ADG with weight gain between D56 and D84 tending to be greater for heifers supplemented with ATX (14.5 kg/period) and BX (13.7 kg/period; *p* = 0.12). It is possible that the performance effects of tannin-based feed additives would be more pronounced if the study was conducted past 84 d. However, performance results should be interpreted carefully as the numerical weight-gain differences between BX and SH and ATX and SH indicate an insufficient number of pasture replicates.

### 3.5. Rectal Temperature, Fecal Scores and Moisture, and Hair Coat Scores

#### 3.5.1. Rectal Temperature

As previously discussed, EA diminishes peripheral blood flow, impairing heat dissipation and increasing internal body temperatures [40]. Tannins may mitigate these effects by binding and inactivating EA. Rectal temperature was numerically greater in control (SH) cattle throughout the study (Table 5).

Additionally, rectal temperature was greater for the SH treatment group on D0 (*p* = 0.08) and D56 (*p* ≤ 0.01). Similarly to the noted weight differences on D0, differences in rectal temperature cannot be attributed to tannin supplementation as cattle were not yet on treatment. On the morning of D56 when cattle were weighed, a weather front had moved through bringing precipitation, wind, and cooler air temperatures which may have contributed to rectal temperature differences. However, it is possible that inclusion of the tannin-based feed additives BX (38.8 °C) and ATX (39.0 °C) may have led to reduced rectal temperatures compared to the SH treatment group (39.1 °C). In the aforementioned study by Poudel et al. [27] lower rectal temperatures for steers receiving *Sericea lespedeza*-based pellets were observed compared to steers receiving *Sericea lespedeza*-based pellets with polyethylene glycol. Similarly, Villalba et al. [17], reported decreased rectal temperatures of lambs grazing wild-type E+ tall fescue that were also consuming the tannin-rich legume sainfoin in contrast to lambs that were consuming the non-tannin-containing legume cicer milkvetch (*Astragalus cicer*).

#### 3.5.2. Fecal Staining Scores and Fecal Moisture

Tannins have been associated with firmer manure and when included in low to moderate amounts, may improve moisture and protein retention [41]. As tannins bind to proteins, this may reduce ruminal ammonia, leading to a shift in N excretion from urine to feces. This impacts overall fecal moisture content as more N is excreted in a solid form (microbial protein and feed particles), leading to a decrease in moisture content [37,41]. However, higher concentrations may negatively impact both animal intake and fiber digestibility [37]. In this study, manure consistency was assessed by fecal staining scoring and evaluating fecal moisture content. Fecal staining scores were measured by 2 observers on a 5.0-point scale with 1.0 representing no staining and 5.0 being equivalent to majority coverage of the perineal area with feces as described previously [25]. Fecal staining scores had a tau value of 0.62 (*p* ≤ 0.01) which suggests moderate agreement between 2 observers. Fecal staining did not differ throughout the supplementation period with the exception of D0 and D84 fecal staining scores. Fecal staining scores were lower for the SH treatment group on D0 (*p* = 0.08) and were lower than that of the BX treatment group on D84 (*p* = 0.05). Scores did not differ between the BX (3.3) and ATX (2.8) treatment groups on D84. Fecal moisture tended to be greater for heifers receiving the SH treatment as compared to the BX and ATX treatment groups on D28 and D84 (Table 5). This is counter to the results shown with fecal staining scores. Although no differences were noted for fecal staining scores or fecal moisture content on D56, it should be noted that this timepoint was unique as the cattle were cooled from rain-wetted hair coats and wind (Figure 2), possibly influencing fluid turnover on that date. Additionally, the scale described by Petrovski et al. [25] may present both interpretation and analysis challenges due to the variable range of coverage encompassed within a score. This especially holds true in the current study as both the number of animals within pasture replicate as well as animals not being the experimental unit constrain fecal scores to being a continuous response variable as opposed to a categorical variable.

#### 3.5.3. Hair Coat Scores

Research has shown that cattle grazing toxic fescue are known to retain winter coats further into summer months [42,43]. Hair coat scores were not affected by treatment (Table 5) and showed a tau value of 0.52 (*p* ≤ 0.01) which suggested moderate agreement between 2 observers. This differs from the results of Poudel et al. [27] who showed that steers grazing primarily E+ tall fescue that were supplemented with Sericea lespedeza-based pellets tended to have lower hair coat scores (3.6) compared to steers receiving Sericea lespedeza-based pellets with polyethylene glycol (4.2). Although there were no differences in hair coat scores between treatments in the current study, scores for all 3 treatments were similar to hair coat scores of steers receiving Sericea lespedeza-based pellets in the study conducted by Poudel et al. [27]. In the current study, hair coat scores averaged 3.4 on a 5.0-point scale at the conclusion of study indicating that heifers, no matter the treatment group, were only 50% shed by the end of June. Although hair coat scores were numerically similar to hair scores of cattle receiving *Sericea lespedeza*-based pellets in the study conducted by Poudel et al. [27], it should be noted that the study by Poudel et al. [27] was conducted at a different time of the yr (late June to mid-September) than the current study, which could impact hair shedding and subsequent hair coat scores.

### 3.6. Serum Analysis Responses

#### 3.6.1. BHV-1 Titers and Haptoglobin

Vaccine-induced neutralizing antibody titers provide an indication of immune protection [44] and vaccine efficacy in cattle [45,46]. Studies suggest that fescue toxicosis may contribute to immunosuppression in cattle through an increase in proinflammatory cytokine secretion [47,48]. This is further purported by anecdotal research suggesting that cattle with tall fescue toxicosis may not respond to vaccination and may experience an increased incidence of bovine respiratory disease complex when shipped to feedlots [49]. It has been hypothesized that decreased prolactin secretion may contribute to humoral immunocompetence as prolactin receptors are present on lymphocytes [49,50] leading to a lower titer response after vaccination [51]. In the current study, we can only speculate on these relationships as we did not collect prolactin samples across multiple timepoints. Dew et al. [51] indicated that rats fed an E+ fescue diet had lower titers after vaccination. However, a study utilizing calves that received a diet supplemented with E+ fescue seed produced similar titers as calves fed endophyte-free tall fescue seed [52]. Tannins have been shown to act as an immunostimulant [53], with dietary inclusion enhancing cell-mediated immune responses in sheep and cattle [53,54,55]. However, there is little research evaluating tannin inclusion in cattle consuming E+ tall fescue on animal immune response.

In the current study, serum antibody titers were present in heifers on D56 (D0 pre-vaccine) indicating that heifers had prior exposure to BHV-1. Titer responses numerically increased after vaccination on D14 post-vaccination (D70 supplementation) which was to be expected (Table 6).

Additionally, there were no treatment differences noted for haptoglobin (*p* = 0.26) on D14 post-vaccination. Haptoglobin values for heifers receiving the ATX treatment were numerically lower compared to SH and BX. In healthy cattle, serum haptoglobin is typically either non-detectable or present in very low concentrations, generally below 10.0 mg/dL. In the current study, haptoglobin values for all treatments were <10 mg/dL, indicating that at this point in time, heifers were healthy or not under stressful conditions.

Titer responses did not differ based on treatment nor were there differences in titer responses between D0–D14 and D14–D28 (*p* > 0.15) post-vaccination. Numerically, titer responses for all treatments declined between D14 and D28 post-vaccination. Results of this study slightly differ from those of Poole et al. [48]. Poole et al. [48] evaluated the impacts of supplemental protein and EA exposure on both adaptive and innate immune responses of vaccinated stocker steers. Blood samples were collected and analyzed for serum cytokine concentrations as well as antibody titers for bovine viral diarrhea virus (BVDV-1b) and IBR virus using a VN test. Steers were vaccinated on D28 of the study and received a booster on D42. Similarly to the results of the current study, titers for IBR did not differ on D0. Additionally, results did not differ on D56. However, the titer response on D42 was greater for steers consuming endophyte-infected seed at 18.0% CP (1.78) compared to the titer response of steers consuming endophyte-free seed at 18.0% CP (0.22). Titers for neither treatment at the 18.0% CP level differed from titers for the endophyte-free and endophyte-infected treatments with 14.0% CP. Poole et al. [48] indicated that steers potentially mounted an overall antibody response to vaccination as circulating antibody levels were below 3.12 (log_2_; [56]). However, levels in this study did not fall below this threshold. It should be noted that while there were not significant differences in titer responses, general antibody responses to vaccination were similar to those observed by Spore et al. [57] and Justice [58].

#### 3.6.2. Blood Urea Nitrogen

Dietary tannins may form stable complexes with proteins within the rumen, reducing BUN formation and urinary N excretion [37,59]. There were no differences between treatments for BUN (*p* = 0.38; Table 6). Blood urea nitrogen values for heifers receiving the ATX treatment were numerically lower (20.8 mg/dL) compared to heifers receiving the SH (24.3 mg/dL) and BX (21.5 mg/dL) treatments. In grazing cattle, a normal BUN range is generally considered to be between 7.0 and 21.0 mg/dL [60]. A study by Brown et al. [61] evaluated serum metabolites of growing steers grazing high or low E+ tall fescue. Blood urea nitrogen concentrations did not differ between treatment groups (16.4 mg/100 mL v. 16.2 mg/100 mL) and fell into the aforementioned normal BUN range. A study by Jackson et al. [62] found similar results with similar BUN values. The authors concluded that because BUN values did not differ between treatments, then hepatic urea synthetic capacities likely did not differ. However, when comparing these results to those of the current study interpretations should be made carefully as tannins were not utilized in the study by Brown et al. [61]. While tannins can bind to and potentially neutralize EA, depending on the amount and type fed, they may also negatively impact protein digestion and N absorption due to the complexes tannins can form with rumen microbes and proteins [63]. Elevated BUN concentrations as seen in this study might indicate increased protein breakdown and inefficient N use. Mean and D84 forage CP tended to be lower (*p* = 0.14) when compared to BX pastures. This likely contributed to numerically lower BUN values, potentially due to less N intake or better protein utilization. The relatively higher CP for the BX treatment may have contributed to greater N excretion.

#### 3.6.3. Serum Prolactin

Prolactin plays a critical role in the shedding of winter coats [27] with reduction in prolactin concentrations being a hallmark symptom of tall fescue toxicosis [64]. It is thought that EA inhibit prolactin synthesis and release, leading to the aforementioned reduction seen in animals grazing E+ tall fescue [64]. In this study, there were no differences between treatments observed for serum prolactin (*p* = 0.97; Table 6). Numerically, serum prolactin was greatest for heifers assigned to the SH treatment (72.3 ng/mL) as compared to heifers assigned to the BX treatment (67.9 ng/mL) and the ATX treatment (71.9 ng/mL). Because samples were only collected on the final d of the study, we cannot determine whether prolactin concentrations decreased or increased during the treatment period. However, as winter hair coat scores were only 50% shed towards the end of the 84 d treatment period, it is possible that prolactin was impacted by ingestion of E+ tall fescue as EA from E+ tall fescue inhibit the shedding of winter hair coats [42]. Results should be interpreted carefully as this was a preliminary study with samples for prolactin only collected on the final d of the study.

### 3.7. Study Limitations

Further research is needed to validate results and would include increasing pasture replicates, animal numbers, and serum samples as well as accounting for experimental variation such as cattle sex, environmental factors, and dosage and type tannin-based feed additives utilized. Additionally, future studies should include an adaptation period to a diet including tannin-based feed additives prior to the beginning of the study to help minimize variability in animal responses due to adaptation challenges as well as allowing for rumen microbes to adapt to the new diet, leading to a stable, more representative microbial population [65,66]. Furthermore, evaluating and identifying the potential underlying mechanisms between EA and tannin-based feed additives have potential broad applications to both producers and researchers utilizing E+ tall fescue pastures as part of their grazing management. Finally, while this study provides initial insights into potential biological effects of tannin supplementation, no cost–benefit analysis was conducted. Given the lack of economic data collected, further research is warranted to assess financial feasibility of implementing tannin-based feed additives in a pasture-based setting.

## 4. Conclusions

While observed effects were modest and limited to a single 28 d period, the findings of this study indicate that dietary inclusion of tannin-based feed additives may contribute to improved heifer ADG and weight gain. The tannin-based feed additive ATX is of particular interest for future studies as numerical differences and trends indicate that ATX may improve heifer performance compared to SH only and BX. However, due to the limited duration and magnitude of observed differences, additional studies with greater replication of a longer duration are needed.

## Figures and Tables

**Table 1:** Hair coat score scale used to evaluate yearling beef heifers grazing toxic, endophyte-infected tall fescue pastures supplemented with soybean hulls (SH) only or SH with commercial dietary tannin-based feed additives between April and June 2024.

Score ^1^	Description
1.0	Smooth short hair coat over entire body (summer coat)
2.0	Rough hair coat over 25% of body
3.0	Rough hair coat over 50% of body
4.0	Rough hair cover 75% of body
5.0	Rough hair coat over entire body (winter coat)

^1^ Adapted from Coffey et al. [7].

**Table 2:** Fecal staining score scale used to evaluate yearling beef heifers grazing toxic, endophyte-infected tall fescue pastures supplemented with soybean hulls (SH) only or SH with commercial dietary tannin-based feed additives between April and June 2024.

Score ^1^	Description ^2^
1.0	No fecal staining (0%)
2.0	Mild; Few flecks of perineal staining (2–10%)
3.0	Moderate; Up to 30% of perineal area stained (11–30%)
4.0	Severe; Large portion of the perineal area stained (31–60%)
5.0	Very severe; Nearly whole perineal area stained (≥60%)

^1^ Adapted from Petrovski et al. [25]. ^2^ Perineal area refers to the area around the anus, caudal hindlimb, rump, and tail.

**Table 3:** Forage mass and nutritive quality of toxic, endophyte-infected tall fescue pastures grazed by yearling beef heifers receiving soybean hulls (SH) only or SH with commercial dietary tannin-based feed additives between April and June 2024.

	Treatments ^1^				
Item	SH	BX	ATX	SEM	*p*-Value
Forage mass, kg/ha					
D0	1612.0	1700.0	1689.0	129.0	0.78
D84	3039.0	3177.0	3064.0	130.0	0.72
Mean ^2^	2153.0 ^d^	2278.0 ^c^	2165.0 ^d^	321.0	0.12
CP, % DM ^3^					
D0	15.6	16.2	14.3	0.70	0.25
D84	10.0 ^c,d^	10.8 ^c^	9.0 ^d^	0.48	0.14
Mean	12.4 ^c,d^	13.0 ^c^	11.7 ^d^	1.23	0.14
ADF, % DM ^4^					
D0	27.2	26.6	26.9	0.37	0.50
D84	38.6	34.3	36.8	2.46	0.49
Mean	33.2	31.9	33.0	2.14	0.44
NDF, % DM ^5^					
D0	47.4 ^a^	45.5 ^b^	47.3 ^a^	0.86	0.06
D84	59.3 ^d^	60.2 ^c,d^	61.7 ^c^	0.63	0.12
Mean	55.4 ^d^	55.5 ^d^	57.1 ^c^	3.23	0.14
TDN, % DM ^6^					
D0	63.1	64.0	61.9	0.80	0.29
D84	52.5	55.1	52.3	1.09	0.24
Mean	57.2	58.4	56.6	2.09	0.16

^1^ All cattle grazed toxic, endophyte-infected tall fescue pastures with treatments consisting of: (1) soybean hulls (SH), (2) SH and the BX additive (10.0 g heifer^−1^ d^−1^; BX), or (3) SH and the ATX additive (5.0 g heifer^−1^ d^−1^; ATX). ^2^ Mean values were calculated based on values collected on D0, D28, D56, and D84. ^3^ Crude protein. ^4^ Acid detergent fiber. ^5^ Neutral detergent fiber. ^6^ Total digestible nutrients. ^a,b^ Within row, means with different superscripts differ at *p* ≤ 0.10. ^c,d^ Within row, means with different superscripts indicate trends at 0.10 < *p* ≤ 0.15.

**Table 4:** Performance of yearling beef heifers grazing toxic, endophyte-infected tall fescue pastures supplemented with soybean hulls (SH) only or SH with commercial dietary tannin-based feed additives between April and June 2024.

	Treatments ^1^				
Item	SH	BX	ATX	SEM	*p*-Value
Weight, kg					
D0	287.1 ^c^	294.4 ^d^	296.6 ^d^	2.6	0.12
D28	309.4	318.0	321.6	6.4	0.63
D56	322.5	325.7	336.6	9.6	0.62
D84	327.9	339.3	352.0	11.6	0.55
ADG, kg/d ^2^					
D0–D28	0.59	0.86	0.93	0.17	0.74
D28–D56	0.44	0.28	0.55	0.10	0.27
D56–D84	0.23 ^a^	0.49 ^b^	0.53 ^b^	0.09	0.07
D0–D84	0.47	0.46	0.67	0.10	0.40
Weight Gained (kg)/Period					
D0–D28	20.7	21.1	25.9	4.8	0.74
D28–D56	12.2	7.6	15.1	2.8	0.25
D56–D84	6.4 ^c^	13.7 ^d^	14.5 ^d^	2.6	0.12
D0–D84	39.1	45.4	56.0	8.4	0.41

^1^ All cattle grazed toxic, endophyte-infected tall fescue pastures with treatments consisting of: (1) soybean hulls (SH), (2) SH and the BX additive (10.0 g heifer^−1^ d^−1^; BX), or (3) SH and the ATX additive (5.0 g heifer^−1^ d^−1^; ATX). ^2^ Average daily gain. ^a,b^ Within row, means with different superscripts differ at *p* ≤ 0.10. ^c,d^ Within row, means with different superscripts indicate trends at 0.10 < *p* ≤ 0.15.

**Table 5:** Rectal temperature, fecal staining scores and moisture, and hair coat scores of yearling beef heifers grazing toxic, endophyte-infected tall fescue pastures supplemented with soybean hulls (SH) only or SH with commercial dietary tannin-based feed additives between April and June 2024.

	Treatments ^1^				
Item	SH	BX	ATX	SEM	*p*-Value
Rectal Temperature, °C					
D0	39.8 ^a^	39.3 ^b^	39.2 ^b^	0.27	0.08
D28	40.3	40.1	40.1	0.35	0.72
D56	39.1 ^a^	38.8 ^c^	39.0 ^b^	0.25	≤0.01
D84	40.5	40.2	40.0	0.40	0.32
Fecal Staining Scores					
D0	2.6 ^a^	3.1 ^b^	3.2 ^b^	0.26	0.08
D28	2.5	1.7	1.8	0.33	0.41
D56	1.3	1.4	1.4	0.32	0.95
D84	2.1 ^a^	3.3 ^b^	2.8 ^a,b^	0.37	0.05
Fecal Moisture, %					
D0	88.6	89.3	90.1	0.62	0.30
D28	91.0 ^a^	89.9 ^b^	89.8 ^b^	0.38	0.14
D56	90.7	90.5	90.5	0.22	0.73
D84	91.1 ^a^	90.1 ^b^	90.5 ^b^	0.31	0.15
Hair Coat Scores					
D0	5.0	5.0	4.9	0.05	0.19
D28	4.6	4.7	4.4	0.11	0.40
D56	4.0	3.8	4.0	0.34	0.82
D84	3.4	3.6	3.3	0.21	0.71

^1^ All cattle grazed toxic, endophyte-infected tall fescue pastures with treatments consisting of: (1) soybean hulls (SH), (2) SH and the BX additive (10.0 g heifer^−1^ d^−1^; BX), or (3) SH and the ATX additive (5.0 g heifer^−1^ d^−1^; ATX). ^a–c^ Within row, means with different superscripts differ at *p* ≤ 0.10.

**Table 6:** Serum metabolites and responses of yearling beef heifers grazing toxic, endophyte-infected tall fescue pastures supplemented with soybean hulls (SH) only or SH with commercial dietary tannin-based feed additives between April and June 2024.

	Treatments ^1^				
Item	SH	BX	ATX	SEM	*p*-Value
BHV-1 VN, log_2_ ^2^					
D0	3.6	3.4	3.1	0.33	0.50
D14	7.7	7.7	7.9	0.20	0.70
D28	7.2	7.4	7.6	0.18	0.29
D14–D0	4.0	4.2	4.8	0.33	0.29
D28–D14	−0.5	−0.3	−0.3	0.17	0.55
Serum Metabolites					
Haptoglobin, log(HP), ng/mL ^3^	7.04	7.27	6.38	0.35	0.26
BUN, mg/dL ^4^	24.3	21.5	20.8	1.74	0.38
Prolactin, ng/mL ^5^	72.3	67.9	71.9	12.9	0.97

^1^ All cattle grazed toxic, endophyte-infected tall fescue pastures with treatments consisting of: (1) soybean hulls (SH), (2) SH and the BX additive (10.0 g heifer^−1^ d^−1^; BX), or (3) SH and the ATX additive (5.0 g heifer^−1^ d^−1^; ATX). ^2^ Samples for infectious bovine rhinotracheitis (BHV-1) were collected on D0, D14, and D28 post-vaccination and corresponded to D56, D70, and D84 of supplementation. ^3^ Haptoglobin samples were collected on D14 following vaccination and corresponded to D70 of supplementation. ^4^ Blood urea nitrogen (BUN) samples were collected on D56 of supplementation. ^5^ Prolactin samples were collected on D84 of supplementation.

## Data Availability

Data that supports the findings of this study are available from the authors upon request.

## Abbreviations

The following abbreviations are used in this manuscript in alphabetical order:

ADF	Acid detergent fiber
ADG	Average daily gain
ATX	SH and the ATX additive (5.0 g heifer^−1^ d^−1^)
BRD	Bovine respiratory disease
BHV-1	Bovine herpesvirus type I
BRSV	Bovine respiratory syncytial virus
BUN	Blood urea nitrogen
BVD	Bovine viral diarrhea
BVDV-1b	Bovine viral diarrhea virus type 1b
BX	SH and the BX additive (10.0 g heifer^−1^ d^−1^)
BW	Body weight
CI	Confidence intervals
CP	Crude protein
CT	Condensed tannins
D	Day(s)
DM	Dry matter
DMI	Dry matter intake
E+	Toxic, endophyte-infected
EA	Ergot alkaloid(s)
EPG	Eggs per gram
FEC	Fecal egg count
HT	Hydrolysable tannins
IBR	Infectious bovine rhinotracheitis
LFST	Livestock and Forestry Research Station
NDF	Neutral detergent fiber
PI3	Parainfluenza3
PI-BVD	Persistent infection with bovine viral diarrhea virus
PSM	Plant secondary metabolite(s)
RIA	Radioimmunoassay
SEM	Standard error of the mean
SH	Soybean hull(s)
TDN	Total digestible nutrients
VN	Virus neutralization
Wk	Week(s)
Yr	Year(s)
